# Prescribed Opioid Dosages, Payer Type, and Self-Reported Outcomes After Surgical Procedures in Michigan, 2018-2020

**DOI:** 10.1001/jamanetworkopen.2023.22581

**Published:** 2023-07-10

**Authors:** Christopher J. Breuler, Christina Shabet, Lia D. Delaney, Craig S. Brown, Yen-Ling Lai, Chad M. Brummett, Mark C. Bicket, Michael J. Englesbe, Jennifer F. Waljee, Ryan A. Howard

**Affiliations:** 1 Department of Surgery, Michigan Medicine, Ann Arbor; 2 University of Michigan Medical School, Ann Arbor; 3 Division of General Surgery, Stanford Medicine, Palo Alto, California; 4 Michigan Opioid Prescribing Engagement Network, Institute for Healthcare Policy and Innovation, University of Michigan, Ann Arbor; 5 Michigan Surgical Quality Collaborative, Ann Arbor; 6 Center for Health Outcomes and Policy, Michigan Medicine, Ann Arbor; 7 Department of Anesthesiology, Michigan Medicine, Ann Arbor

## Abstract

**Importance:**

Collaborative quality improvement (CQI) models, often supported by private payers, create hospital networks to improve health care delivery. Recently, these systems have focused on opioid stewardship; however, it is unclear whether reduction in postoperative opioid prescribing occurs uniformly across health insurance payer types.

**Objective:**

To evaluate the association between insurance payer type, postoperative opioid prescription size, and patient-reported outcomes in a large statewide CQI model.

**Design, Setting, and Participants:**

This retrospective cohort study used data from 70 hospitals within the Michigan Surgical Quality Collaborative clinical registry for adult patients (age ≥18 years) undergoing general, colorectal, vascular, or gynecologic surgical procedures between January 1, 2018, and December 31, 2020.

**Exposure:**

Insurance type, classified as private, Medicare, or Medicaid.

**Main Outcomes and Measures:**

The primary outcome was postoperative opioid prescription size in milligrams of oral morphine equivalents (OME). Secondary outcomes were patient-reported opioid consumption, refill rate, satisfaction, pain, quality of life, and regret about undergoing surgery.

**Results:**

A total of 40 149 patients (22 921 [57.1%] female; mean [SD] age, 53 [17] years) underwent surgery during the study period. Within this cohort, 23 097 patients (57.5%) had private insurance, 10 667 (26.6%) had Medicare, and 6385 (15.9%) had Medicaid. Unadjusted opioid prescription size decreased for all 3 groups during the study period from 115 to 61 OME for private insurance patients, from 96 to 53 OME for Medicare patients, and from 132 to 65 OME for Medicaid patients. A total of 22 665 patients received a postoperative opioid prescription and had follow-up data for opioid consumption and refill. The rate of opioid consumption was highest among Medicaid patients throughout the study period (16.82 OME [95% CI, 12.57-21.07 OME] greater than among patients with private insurance) but increased the least over time. The odds of refill significantly decreased over time for patients with Medicaid compared with patients with private insurance (odds ratio, 0.93; 95% CI, 0.89-0.98). Adjusted refill rates for private insurance remained between 3.0% and 3.1% over the study period; adjusted refill rates among Medicare and Medicaid patients decreased from 4.7% to 3.1% and 6.5% to 3.4%, respectively, by the end of the study period.

**Conclusions and Relevance:**

In this retrospective cohort study of surgical patients in Michigan from 2018 to 2020, postoperative opioid prescription size decreased across all payer types, and differences between groups narrowed over time. Although funded by private payers, the CQI model appeared to have benefitted patients with Medicare and Medicaid as well.

## Key Points

**Question:**

Is there an association between health insurance payer type and postoperative opioid prescribing in a statewide, privately funded collaborative quality improvement (CQI) model?

**Findings:**

In this cohort study of 40 149 patients undergoing common surgical procedures in a CQI model in Michigan from 2018 to 2020, postoperative opioid prescribing decreased across all payer types, and differences in opioid prescribing between payer types narrowed over time.

**Meaning:**

The findings suggest that a privately funded CQI model standardizing opioid prescribing benefits patients with Medicare, Medicaid, and private insurance and that postoperative opioid prescribing became standardized over time between insurance types.

## Teaser

This cohort study examines associations between postoperative opioid prescribing, patient-reported outcomes, and insurance payer type in a privately funded collaborative quality improvement model in Michigan.

## Introduction

Recently, collaborative quality improvement (CQI) models have emerged in an effort to achieve rapid and sustainable improvements in the care of surgical patients.^1,2,3,4^ These programs, funded by large private payers, engage participating hospitals as a learning health system and create registries of clinical data used to guide quality improvement.^5^ For example, privately funded CQI models have enhanced perioperative processes of care, such as venous thromboembolism chemoprophylaxis, bowel preparation prior to colectomy, and surgical site infection prophylaxis.^6,7,8^ As a result, CQI models have been associated with significantly decreased morbidity among patients undergoing common surgical procedures and with improved patient experience and have optimized the cost and value of surgical interventions.^9,10,11^

Collaborative quality improvement models have been similarly used to reduce postoperative opioid overprescribing. For decades, opioids have been commonly overprescribed following surgery, resulting in leftover pills available for diversion and misuse.^12,13,14,15,16,17,18,19^ Opioid prescribing guidelines developed and implemented using privately funded CQI models in Michigan have led to decreased physician overprescribing and patient consumption while maintaining pain control and satisfaction among patients.^20,21,22^ These changes are incentivized, in part, by additional reimbursement for surgeon adherence to prescribing guidelines, but this additional reimbursement applies only to patients with private insurance.^20,21,22^ Despite the success of the CQI model, the extent to which gains in health care quality achieved by this model extend across different health insurance payer types is unclear. This is concerning given known discrepancies in opioid prescribing and morbidity across payer types.^23,24,25^ Furthermore, in Michigan, 38% of individuals are covered by state or federal health insurance plans and 6% are uninsured.^26^ A uniform benefit to all patients regardless of payer type is imperative if CQI models are to be an effective strategy to address the ongoing opioid epidemic. Uniform prescribing recommendations also present the opportunity to decrease overprescribing by health care practitioners, thus reducing potential harm to the patient and to the community.^27^

Within this context, we performed a retrospective cohort study to examine the association between payer type, postoperative opioid prescribing, and patient-reported outcomes (PROs) after surgery within a privately funded CQI model. We evaluated whether changes in opioid prescribing were similar among patients with employer-based (private) insurance, Medicare, and Medicaid coverage. We also investigated whether PROs varied between these groups. We hypothesized that opioid prescribing would decline at the same rate over time and that PROs would remain similar across all health insurance payer types.

## Methods

### Study Design and Patient Selection

This cohort study of deidentified data was approved by the institutional review board at the University of Michigan; informed consent was not obtained due to the institution’s policy for using deidentified patient data. The study followed the Strengthening the Reporting of Observational Studies in Epidemiology (STROBE) reporting guideline.^28^ We performed a retrospective review of the Michigan Surgical Quality Collaborative (MSQC) clinical registry, a statewide CQI involving 70 hospitals in the state of Michigan at the time of data collection and funded by the largest commercial payer in the state. The MSQC maintains a registry of clinical data for patients undergoing surgical procedures and represents all hospitals that perform major surgery in Michigan.^1^ Data are collected by trained nurse abstractors and include demographics, surgical admission data, preoperative comorbidities, intraoperative details, and 30-day postoperative outcomes. Patient-reported outcomes, including opioid consumption and opioid refills, are collected as part of a follow-up survey administered via telephone, email, or postal mail between postdischarge days 30 and 90. Cases are sampled using an algorithm designed to minimize selection bias, and data are audited annually for accuracy.^29^ Surgical best practices and recommendations developed using MSQC data are disseminated at regular quarterly meetings and on the MSQC website.^20,22^

The CQI prescribing guidelines were released to hospitals in October 2017. We examined opioid-naive adult patients (age ≥18 years) who underwent surgical procedures between January 1, 2018, and December 31, 2020, immediately following CQI implementation, which allowed for assessment at the beginning of the initiative and the ability to follow the initiative as it progressed. Opioid-naive status was defined as no opioid use in the year prior to surgery per medical record review by trained nurse abstractors. Procedure categories included laparoscopic cholecystectomy, laparoscopic appendectomy, laparoscopic and open colectomy, minor hernia repair (inguinal and umbilical), major hernia repair (ventral and incisional), laparoscopic hysterectomy, vaginal hysterectomy, and total abdominal hysterectomy. All procedure categories had evidence-based prescribing guidelines developed, which were previously shown to lead to decreases in postoperative opioid prescription size.^20^ These were intentionally selected to allow for identification of heterogeneity among health insurance payer types across procedures. Only patients with private, Medicare, or Medicaid insurance were included. Patients were excluded from this study if they died within 30 days of surgery, had a length of stay more than 30 days, were discharged to a facility other than home, or had any missing or invalid data.

### Explanatory Variables

Our key explanatory variable was payer type. To evaluate postoperative opioid prescribing and PROs across payer types, patients were grouped into the following categories: private insurance, Medicare, and Medicaid.^30^ Private insurance included any commercial health plan or health maintenance organization (HMO) plan. Medicare included Medicare, Medicare with a supplemental plan such as Medigap, or a Medicare Advantage plan. Medicaid included standard Medicaid or a Medicaid HMO plan. Medicare and Medicaid dual enrollees were not included in the final analysis due to low prevalence (591 patients).

Patient characteristics of interest included age, sex, race and ethnicity, American Society of Anesthesiologists (ASA) classification, obesity, cancer, tobacco use in the year prior to surgery, diabetes, chronic obstructive pulmonary disease, congestive heart failure, hypertension, long-term steroid use, kidney compromise (defined as requiring dialysis within the 2 weeks prior to surgery), and functional status (independent vs not independent). Race and ethnicity were ascertained by trained nurse abstractors through review of medical records and were included in the analysis to understand the demographics of the patient population and adjust for any differences in outcomes that may be influenced by race or ethnicity; categories included Hispanic, non-Hispanic Black, non-Hispanic White, and other (American Indian or Alaska Native, Asian, or Native Hawaiian or Pacific Islander individuals who were not Hispanic or whose Hispanic status was unknown as well as individuals whose race and ethnicity were unknown). The ASA classification is defined by the MSQC as the patient’s present physical condition on a scale of 1 to 5 as it appears on the anesthetic record, with a higher score indicating the presence of increasingly severe systemic disease. Long-term steroid use was defined as regular use of an oral or parenteral corticosteroid for a chronic medical condition within 30 days prior to surgery. Clinical characteristics included admission status (inpatient vs outpatient), surgical priority (elective vs urgent or emergent), surgical approach (open vs minimally invasive), and procedure type.

### Outcomes

Our primary outcome of interest was initial postoperative opioid prescription size, defined as the first prescription written by a health care practitioner postoperatively. Opioid prescription size was calculated as the number of pills multiplied by the dose of each pill in milligrams of oral morphine equivalent (OME) to adjust for varying potencies between different opioid medication types.^31^

Secondary outcomes of interest were PROs, including opioid consumption, opioid prescription refill, satisfaction with care, regret about undergoing surgery, quality of life, and pain in the week after surgery. Opioid consumption and refill were evaluated only for patients who received a postoperative opioid prescription. These secondary outcomes were available for a subset of patients in the overall primary cohort who responded to a survey administered between postoperative days 30 to 90. In the survey, patients were asked to report how many opioid pills they consumed, whether they refilled their opioid prescription, and their PRO measures. Given variation in workflow and workload at participating MSQC hospitals, each site determined how many patients were contacted for postoperative survey follow-up to provide a representative sample of postoperative outcomes.^27^

Satisfaction with care, regret about undergoing surgery, and quality of life were collected using questions adapted from the O’Connor and Holmes-Rovner scales of satisfaction and regret.^32,33^ Responses were highly skewed, and therefore, these outcomes were dichotomized into binary outcomes.^34^ Dichotomization of skewed PRO responses is routinely performed for many Hospital Consumer Assessment of Healthcare Providers and Systems responses.^35,36^ Satisfaction with care was measured on a scale from 0 (extremely dissatisfied) to 10 (extremely satisfied) and dichotomized into “highly satisfied” for scores of 9 or 10 and “not highly satisfied” for scores of 0 to 8. Regret about undergoing surgery was measured on a scale of 1 (absolutely regret surgery) to 5 (absolutely no regret about undergoing surgery) and dichotomized into “absolutely no regret” for a score of 5 and “some regret” for scores of 1 to 4. Quality of life was measured on a scale of 1 (absolute worst quality of life) to 5 (absolute best quality of life) and dichotomized into “absolute best quality of life” for a score of 5 and “less than best quality of life” for scores of 1 to 4. Pain in the week after surgery was measured on a scale of 1 to 4, with 1 indicating no pain; 2, minimal pain; 3, moderate pain; and 4, severe pain.

### Statistical Analysis

Univariate comparisons of descriptive statistics were performed to characterize baseline patient characteristics among different payer types using the χ^2^ test and analysis of variance, as appropriate. Multivariable linear regression models were used to evaluate the association of payer type with initial prescription size and consumption. To account for the violation of homoscedasticity, robust SEs were calculated. Multivariable logistic regression models were used to evaluate the association of payer type with refill, satisfaction with care, regret about undergoing surgery, and quality of life. An ordered logistic regression model was used to analyze the association between payer type and pain. All explanatory covariates were used in these models. The quarter of the year during which the surgery took place was added to the regression models to assess the effect of time or interaction between payer types and time. Orthogonal polynomial trend contrasts were applied to detect whether a linear trend of time existed. Prescription size was used as an additional covariate in the model for opioid consumption given the known association between prescription size and consumption.^27^ Statistical analyses were performed using Stata, version 15.0 (StataCorp LLC), and *P* values were 2-sided with significance set at ≤.05.

## Results

A total of 40 149 patients met inclusion criteria for the primary outcome of prescription size (eFigure 1 in Supplement 1), with the patient cohort having a mean (SD) age of 53 (17) years; 22 921 patients (57.1%) were female; 17 228 (42.9%), male; 956 (2.4%), Hispanic; 3146 (7.8%), non-Hispanic Black; 32 926 (82.0%), non-Hispanic White; and 3121 (7.8%), other race and ethnicity. The demographic attributes of the cohort are presented in Table 1. Within this cohort, 23 097 patients (57.5%) had private insurance, 10 667 (26.6%) had Medicare, and 6385 (15.9%) had Medicaid. Data on satisfaction, pain, quality of life, and regret about undergoing surgery were available for 29 369 patients who responded to a postoperative survey (73.2% of the original cohort). Comparisons between the overall sample and the sample who responded to the postoperative survey are given in eTable 1 in Supplement 1. Significant differences were found for age, ASA classification, smoking status, diagnosis of hypertension, and procedure type. There were no significant differences found in other comorbid conditions between groups. Mean (SD) age of the surveyed cohort was 54 (17) years; 16 597 patients (56.5%) were female, and 12 772 (43.5%) were male. In this group, 16 480 patients (56.1%) had private insurance, 8552 (29.1%) had Medicare, and 4337 (14.8%) had Medicaid. There were 22 665 patients (56.5% of the total cohort) who had received a postoperative opioid prescription and had data for consumption and refill.

**Table 1.** Baseline Cohort Characteristics and Univariate Analysis^a^

Characteristic	Patients, No. (%)		
	Private insurance (n = 23 097)	Medicare (n = 10 667)	Medicaid (n = 6385)
Age, y			
18-29	2515 (10.9)	34 (0.3)	1450 (22.7)
30-39	3849 (16.7)	108 (1.0)	1657 (26.0)
40-49	5802 (25.1)	221 (2.1)	1453 (22.8)
50-59	6392 (27.7	391 (3.7)	1201 (18.8)
60-64	3324 (14.4)	426 (4.0)	533 (8.4)
≥65	1215 (5.3)	9487 (88.9)	91 (1.4)
Sex			
Female	13 500 (58.5)	5053 (47.4)	4368 (68.4)
Male	9597 (41.6)	5614 (52.6)	2017 (31.6)
Race and ethnicity			
Black, non-Hispanic	1395 (6.0)	558 (5.2)	1193 (18.7)
Hispanic	544 (2.4)	111 (1.0)	301 (4.7)
White, non-Hispanic	19 298 (83.6)	9246 (86.7)	4382 (68.6)
Other^b^	1860 (8.1)	752 (7.1)	509 (8.0)
ASA classification			
1	3061 (13.3)	207 (1.9)	528 (8.3)
2	15 238 (66.0)	4642 (43.5)	4002 (62.7)
3	4682 (20.3)	5410 (50.7)	1785 (28.0)
4-5	116 (0.5)	408 (3.8)	70 (1.1)
Obesity	11 208 (48.5)	3960 (37.1)	3367 (52.7)
Cancer	1069 (4.6)	1238 (11.6)	208 (3.3)
Smoking	3525 (15.3)	1174 (11.0)	2515 (39.4)
Diabetes	1733 (7.5)	1944 (18.2)	579 (9.1)
Not independent	19 (0.1)	98 (0.9)	20 (0.3)
Chronic obstructive pulmonary disease	306 (1.3)	743 (7.0)	246 (3.9)
Congestive heart failure	12 (0.1)	47 (0.4)	9 (0.1)
Hypertension	6142 (26.6)	6715 (63.0)	1631 (25.5)
Chronic steroid use	340 (1.5)	282 (2.6)	98 (1.5)
Dialysis	21 (0.1)	51 (0.5)	8 (0.1)
Inpatient admission status	10 401 (45.0)	5309 (49.8)	3270 (51.2)
Urgent or emergent surgical priority	5497 (23.8)	2296 (21.5)	1882 (29.5)
Surgical approach			
Minimally invasive	18 031 (78.1)	7233 (67.8)	5051 (79.1)
Open	5066 (21.9)	3434 (32.2)	1334 (20.9)
Procedure type			
Major hernia	876 (3.8)	601 (5.6)	262 (4.1)
Minor hernia	6418 (27.8)	3988 (37.4)	1514 (23.7)
Laparoscopic appendectomy	3085 (13.4)	599 (5.6)	806 (12.6)
Laparoscopic cholecystectomy	6192 (26.8)	2830 (26.5)	2192 (34.3)
Laparoscopic colectomy	782 (3.4)	801 (7.5)	154 (2.4)
Open colectomy	436 (1.9)	573 (5.4)	118 (1.9)
Vaginal hysterectomy	1357 (5.9)	403 (3.8)	346 (5.4)
Laparoscopic hysterectomy	3006 (13.0)	665 (6.2)	728 (11.4)
Total abdominal hysterectomy	945 (4.1)	207 (1.9)	265 (4.2)

Abbreviation: ASA, American Society of Anesthesiologists.

^a^ *P* < .001 for all comparisons.

^b^ Other includes the following races and ethnicities: American Indian or Alaska Native and not Hispanic, American Indian or Alaska Native and Hispanic status unknown, Asian and not Hispanic, Asian and Hispanic status unknown, Black and Hispanic status unknown, Native Hawaiian or Pacific Islander and not Hispanic, Native Hawaiian or Pacific Islander and Hispanic status unknown, race unknown and not Hispanic, White and Hispanic status unknown, and unknown.

Mean (SD) opioid prescription size over the study period was 75.9 (53.9) OME among patients with Medicaid, 72.0 (52.4) OME among patients with private insurance, and 62.5 (52.3) OME among patients with Medicare (*P* < .001) (Table 2). Mean (SD) consumption was greatest among patients with Medicaid (54.3 [50.2] OME) compared with patients with private insurance (36.0 [40.7] OME) and Medicare (26.8 [35.6] OME) (*P* < .001). Patients with Medicaid also had a higher rate of medication refills at 6.1% (218 of 3595 patients) compared with 3.3% for patients with private insurance (440 of 13 385) and 2.4% for patients with Medicare (137 of 5685). Differences in PROs are presented in Table 2.

**Table 2.** Unadjusted Primary and Secondary Outcomes by Insurance Type

Outcome	Private	Medicare	Medicaid	*P* value
Prescription size, mean (SD), OME^a^	72.0 (52.4)	62.5 (52.3)	75.9 (53.9)	<.001
Consumption size, mean (SD), OME^b^	36.0 (40.7)	26.8 (35.6)	54.3 (50.2)	<.001
Refill, No. (%)^b^	440 (3.3)	137 (2.4)	218 (6.1)	<.001
High satisfaction, No. (%)^c^	13 500 (81.9)	7140 (83.5)	3557 (82.0)	.007
Absolutely no regret about undergoing surgery, No. (%)^c^	11 153 (67.7)	4961 (58.0)	2982 (68.8)	<.001
Best quality of life, No. (%)^c^	15 265 (92.6)	7953 (93.0)	3937 (90.8)	<.001
Pain after surgery, No. (%)^c^				
None	1004 (6.1)	1243 (14.5)	200 (4.6)	<.001
Minimal	7145 (43.4)	3990 (46.7)	1458 (33.6)	
Moderate	6798 (41.3)	2765 (32.3)	1849 (42.6)	
Severe	1533 (9.3)	554 (6.5)	830 (19.1)	

Abbreviation: OME, oral morphine equivalent.

^a^ Data were available for 40 149 patients (23 097 with private insurance, 10 667 with Medicare, and 6385 with Medicaid).

^b^ Data were available for 22 665 patients (13 385 with private insurance, 5865 with Medicare, and 3595 with Medicaid).

^c^ Data were available for 29 369 patients who responded to follow-up surveys (16 480 with private insurance, 8552 with Medicare, and 4337 with Medicaid).

Table 3 details the adjusted outcomes for prescription size, consumption size, refill rate, and PROs. Estimated mean opioid prescription size over the study period was 72.8 OME (95% CI, 71.4-74.2 OME) among patients with Medicaid, 69.9 OME (95% CI, 69.2-70.7 OME) among patients with private insurance, and 68.8 OME (95% CI, 67.3-70.3 OME) among patients with Medicare (*P* < .001) (Table 3). Estimated mean consumption size was also greatest among patients with Medicaid (44.5 OME; 95% CI, 43.1-46.0 OME) compared with patients with private insurance (34.5 OME; 95% CI, 33.8-35.3 OME) and Medicare (36.6 OME; 95% CI, 35.1-38.1 OME) (*P* < .001). Patients with Medicaid also had a higher estimated mean rate of medication refills (4.6%; 95% CI, 4.0%-5.3%) compared with patients with private insurance (3.1%; 95% CI, 2.8%-3.4%) and patients with Medicare (3.8%; 95% CI, 2.8%-4.8%).

**Table 3.** Adjusted Outcomes by Insurance Type^a^

Outcome	Private		Medicare			Medicaid		*P* value^c^
	Estimated mean (95% CI)^b^	SE	Estimated mean (95% CI)^b^	SE	Estimated mean (95% CI)^b^		SE	
Prescription size, OME^d^	69.9 (69.2-70.7)	0.39	68.8 (67.3-70.3)	0.77	72.8 (71.4-74.2)		0.72	<.001
Consumption size, OME^e^^,^^f^	34.5 (33.8-35.3)	0.37	36.6 (35.1-38.1)	0.77	44.5 (43.1-46.0)		0.73	<.001
Refill. %^e^	3.1 (2.8-3.4)	0.16	3.8 (2.8-4.8)	0.51	4.6 (4.0-5.3)		0.34	<.001
High satisfaction, %^g^	82.1 (81.4-82.8)	0.37	82.9 (81.6-84.3)	0.69	82.4 (81.1-83.6)		0.66	.47
Absolutely no regret about undergoing surgery, %^g^	92.9 (92.4-93.3)	0.24	92.0 (91.0-93.0)	0.53	91.8 (90.9-92.7)		0.45	.57
Best quality of life, %^g^	66.2 (65.3-67.1)	0.47	62.7 (61.0-64.4)	0.85	65.5 (63.8-67.1)		0.84	<.001
Pain after surgery, %^g^								
None	8.3 (7.9-8.7)	0.21	8.9 (8.4-9.5)	0.28	6.6 (6.1-7.1)		0.25	.07
Minimal	43.3 (42.6-44.0)	0.35	44.5 (43.3-45.8)	0.64	39.0 (37.8-40.2)		0.60	
Moderate	38.8 (38.0-39.5)	0.37	37.6 (36.5-38.7)	0.57	42.3 (41.3-43.4)		0.54	
Severe	9.6 (9.2-10.0)	0.20	9.0 (8.3-9.7)	0.34	12.0 (11.4-12.7)		0.36	

Abbreviation: OME, oral morphine equivalent.

^a^ Adjusted for age, sex, race and ethnicity, American Society of Anesthesiologists classification, obesity, cancer, tobacco use, diabetes, functional status, chronic obstructive pulmonary disease, congestive heart failure, hypertension, long-term steroid use, dialysis, admission status, surgical priority, surgical approach, procedure type, time (quarter of the year), and insurance group × time interaction.

^b^ Estimated means were averaged across all time points.

^c^ *P* values are for the difference between the 3 insurance groups.

^d^ Data were available for 40 149 patients (23 097 with private insurance, 10 667 with Medicare, and 6385 with Medicaid).

^e^ Data were available for 22 665 patients (13 385 with private insurance, 5865 with Medicare, and 3595 with Medicaid).

^f^ Consumption size was also adjusted for prescription size.

^g^ Data were available for 29 369 patients who responded to follow-up surveys (16 480 with private insurance, 8552 with Medicare, and 4337 with Medicaid).

### Opioid Prescription Size

**Figure 1.**
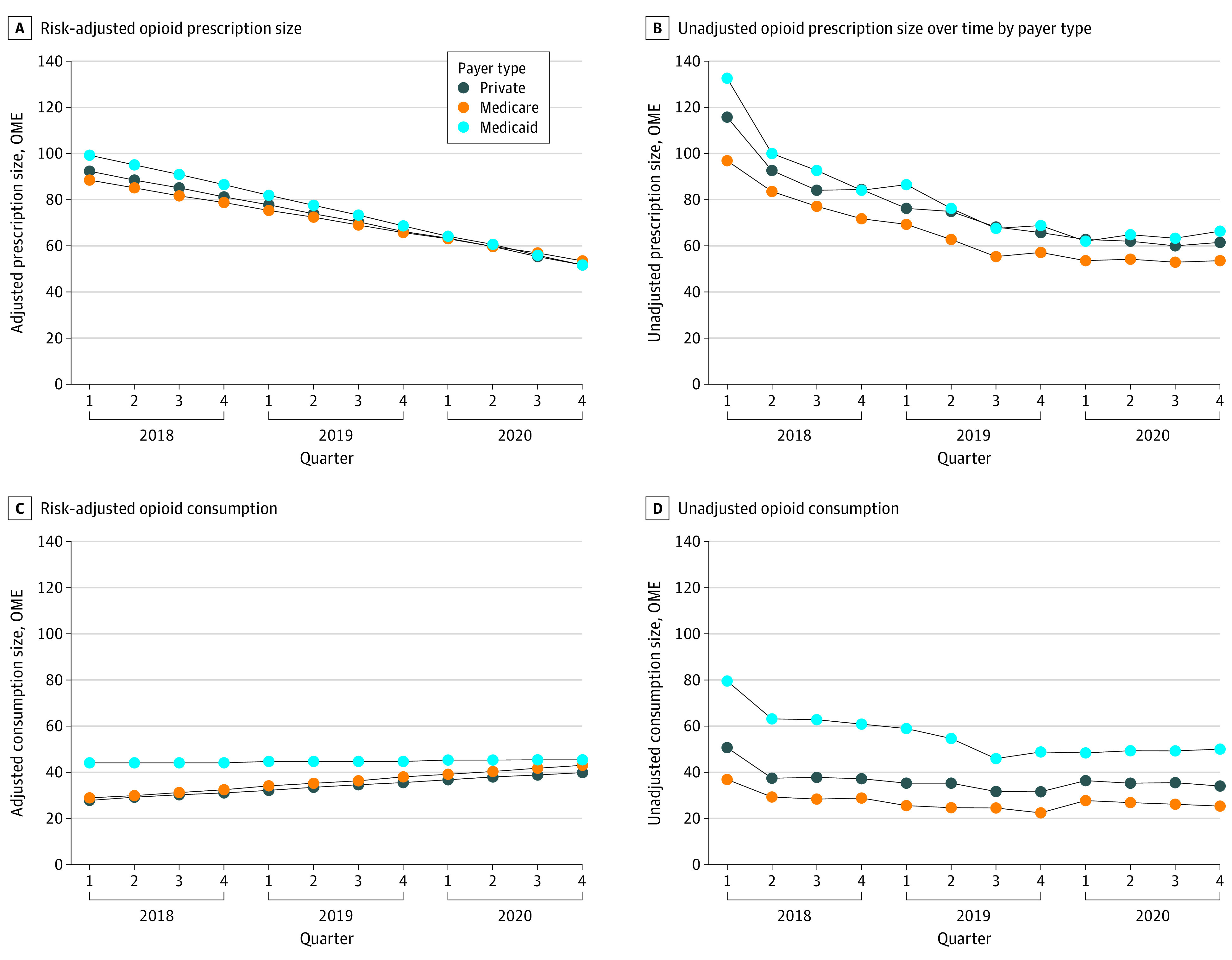
Risk-Adjusted and Unadjusted Opioid Prescription Size and Consumption Over Time by Payer Type OME indicates oral morphine equivalent.

Payer type was significantly associated with prescription size, and Medicaid patients received prescriptions that were on average 7.68 OME (95% CI, 3.59-11.78 OME) larger than those of patients with private insurance. Notably, prescription size declined differently over time by payer type, with faster declines among Medicaid patients by an additional 0.68 OME (95% CI, 0.20-1.15 OME) on average per quarter compared with patients with private insurance. Orthogonal polynomial trend contrasts of prescription size over time fit a linear trend. Figure 1A details adjusted prescription size over time by payer type, and Figure 1B details unadjusted opioid prescription size over time by payer type. Unadjusted prescription size decreased from 115 to 61 OME for patients with private insurance, from 96 to 53 OME for Medicare patients, and from 132 to 65 OME for Medicaid patients over the study period. Adjusted prescription size decreased from 92 OME to 52 OME for privately insured patients, from 88 OME to 53 OME for Medicare patients, and from 99 OME to 52 OME for Medicaid patients.

### Opioid Consumption and Refill

Opioid consumption was also significantly associated with payer type, with Medicaid patients consuming on average 16.82 OME (95% CI, 12.57-21.07 OME) more per quarter than patients with private insurance (equivalent to roughly 2 tablets of oxycodone, 5 mg). However, rates of opioid consumption differed among payer types and decreased among Medicaid patients by 0.96 OME (95% CI, 0.46-1.45 OME) per quarter compared with patients with private payer coverage (Figure 1C and D). Orthogonal polynomial trend contrasts of opioid consumption over time fit a linear trend.

**Figure 2.**
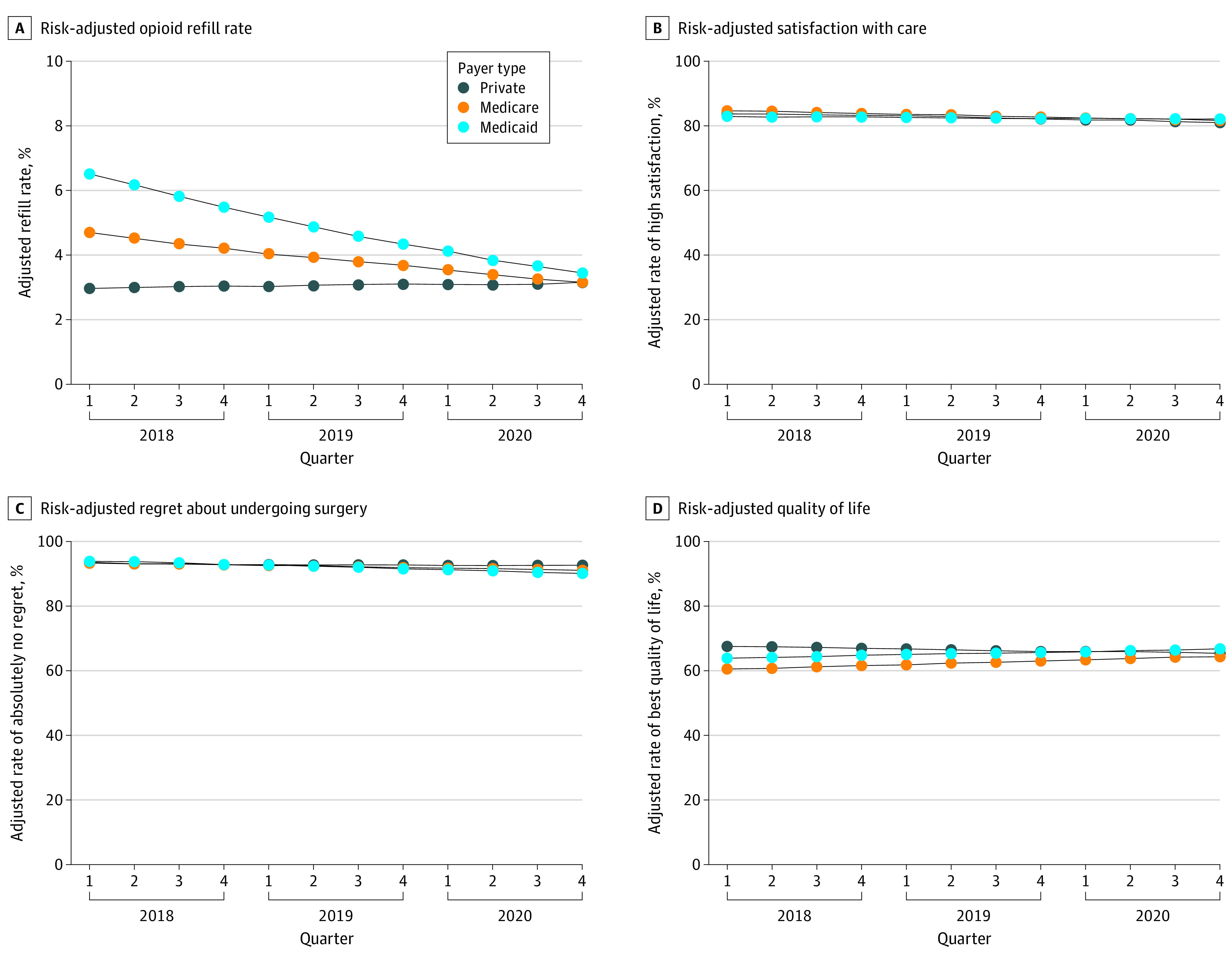
Risk-Adjusted Opioid Refill Rate, Satisfaction, Regret, and Quality of Life

Refill was also significantly associated with payer type, with Medicaid and Medicare patients having higher odds of refill compared with patients with private insurance (Medicaid: odds ratio [OR], 2.46 [95% CI, 1.66-3.67]; Medicare: OR, 1.69 [95% CI, 1.01-2.84]). Orthogonal polynomial trend contrasts of refill over time fit a linear trend. Private insurance adjusted refill rates remained between 3.0% and 3.1% over the study period. Medicare and Medicaid adjusted refill rates decreased from 4.7% to 3.1% and from 6.5% to 3.4%, respectively, mirroring the refill rate among private insurance patients by the end of the study period. Medicaid patients had lower odds of refill over time compared with patients with private insurance (OR, 0.93; 95% CI, 0.89-0.98). Medicare patients did not significantly differ in odds of refill over time compared with patients with private insurance (Figure 2A).

### Satisfaction, Regret, Quality of Life, and Pain

There was no association between satisfaction with care and payer type among Medicare (OR, 1.09; 95% CI, 0.89-1.34; *P* = .41) and Medicaid (OR, 0.92; 95% CI, 0.74-1.15; *P* = .48) patients compared with patients with private insurance. Moreover, satisfaction did not change over time for different payer types, with orthogonal polynomial trend contrasts detecting a linear trend of time (eFigure 2 and eTable 2 in Supplement 1). There was also no significant association between regret and insurance type among Medicare (OR, 1.03; 95% CI, 0.76-1.39; *P* = .85) and Medicaid (OR, 1.19; 95% CI, 0.86-1.64; *P* = .29) patients compared with patients with private insurance. Regret did not change over time for different payer types and also had a linear trend over time identified by orthogonal trend contrast (eFigure 2 and eTable 2 in Supplement 1).

Patient-reported quality of life did not change over time (OR, 0.99; 95% CI, 0.98-1.00; *P* = .09), but differences existed by payer type. Medicare patients were less likely to have best quality of life compared with patients with private insurance (OR, 0.71; 95% CI, 0.61-0.83). There was no significant difference between Medicaid patients and patients with private insurance. Quality of life changed differently over time by payer type for Medicare patients compared with patients with private insurance (OR, 1.03; 95% CI, 1.01-1.04). Medicaid patients did not have significantly higher odds of best quality of life over time compared with patients with private insurance (OR, 1.02; 95% CI, 1.00-1.04) (Figure 2D and eTable 3 in Supplement 1). Orthogonal polynomial trend contrasts of quality of life over time fit a linear trend.

There was no significant association between pain in the week after surgery and payer type among Medicare (OR, 0.88; 95% CI, 0.76-1.02) and Medicaid (OR, 1.10; 95% CI, 0.94-1.30) patients compared with patients with private insurance. Pain changed differently over time in Medicaid patients (OR, 1.02; 95% CI, 1.00-1.04; *P* = .04) compared with patients with private insurance (eTable 3 in Supplement 1). Orthogonal polynomial trend contrasts of pain over time fit a linear trend.

## Discussion

In this retrospective cohort study of a statewide CQI program, postoperative opioid prescription size decreased over time across all payer types. Despite this decrease in prescription size, there were no changes in satisfaction or regret about undergoing surgery. Moreover, differences in opioid consumption and refill rate between payer types narrowed over the study period. Overall, these results suggest that CQI initiatives, which are often funded by private payers, can be considered as a strategy to reduce postoperative opioid overprescribing for all patients regardless of health insurance payer type.

The current study adds to the understanding of privately funded CQI models as a strategy to address postoperative opioid overprescribing. Specifically, this study found that there were benefits for all patients regardless of health insurance payer type, evidenced by a decrease in opioid prescription size across groups. Although additional reimbursement as part of this CQI-sponsored program only applied to patients with private insurance, these findings suggest that these efforts were applied more broadly to all patients, which may simply be more pragmatic for hospitals. Furthermore, we observed a narrowing of differences over time. For example, although postoperative opioid prescription size and refill rate were initially highest for patients with Medicaid, both outcomes declined faster in this group compared with patients with private insurance. Additionally, although consumption was initially highest for patients with Medicaid, differences between groups also narrowed over the study period. As a result, postoperative opioid prescription size, refill rate, and consumption were similar between groups at the end of the study period. In the context of an ongoing opioid epidemic, the need for effective development and dissemination of postoperative prescribing recommendations is imperative.^37^ The homogeneous outcomes of opioid prescribing guidelines across health insurance payer types seen in this study further support the value of privately funded statewide CQIs and suggest that this model may be a useful strategy for addressing the ongoing opioid epidemic in other regions. Moreover, the results of this study may serve as evidence of a CQI framework that could be extrapolated to other health conditions and public health efforts.

While these findings suggest that privately funded CQI initiatives are associated with reduced opioid prescribing across payer types, differences in PROs still existed. Specifically, we found that Medicaid patients had higher patient-reported opioid consumption and opioid refill rates over time compared with patients with private insurance or Medicare. This finding is consistent with previous literature highlighting inequities among Medicaid patients related to opioid prescribing and opioid-related morbidity, although these findings may be a proxy for important unmeasured characteristics among Medicaid patients.^23,24,25^ For example, disability, chronic disease, and surgical morbidity and mortality have historically been higher among Medicaid patients.^30,38,39,40,41^ Medicaid beneficiaries are also more likely to report lower satisfaction and worse pain compared with privately insured patients.^42^ While it is important to consider other socioeconomic confounders as potential contributors to these findings as well as the consequences of out-of-pocket costs and cost-sharing for opioid use, our study suggests that Medicaid patients remain at higher risk for opioid-related harms compared with patients with other payer types despite considerable improvement in opioid outcomes during the study period.^43,44,45,46,47,48^

### Limitations

There are several limitations to this study. First, our PROs may have been affected by response bias given that data were obtained between 30 and 90 days postoperatively during follow-up. However, we observed that the distribution of payer types in our subanalysis was similar to that in the main cohort. Second, because this was a pilot effort, sampling algorithms were decided by each hospital’s clinical nurse reviewer based on feasibility given a fluctuating workload. This resulted in not all patients within the MSQC database being contacted for PRO collection and introduced the possibility of variability within the postoperative survey follow-up cohort. We did not include patients who died after surgery, and not including this small subset of patients may have resulted in not capturing the true rate of long-term adverse events, satisfaction, consumption, and regret. In addition, while opioid prescribing guideline implementation has been shown to lead to decreased opioid prescribing while maintaining patient satisfaction and pain control,^20,21^ some of the observed changes over time in this study might be explained by other competing factors, including legislation, increased education about opioid prescribing, and changing culture around opioids, which were not accounted for in this study.

## Conclusions

In this cohort study of patients in a CQI model in Michigan from 2018 to 2020, postoperative opioid prescription size decreased over time for patients with private, Medicare, and Medicaid insurance, and differences between groups narrowed over the study period. Opioid consumption, refill rate, and quality-of-life measures also converged between insurance groups toward the end of the follow-up period. These findings support the use of a privately funded statewide CQI model to address the opioid epidemic, demonstrating similar benefit to patients regardless of payer type. Future work should investigate whether these models can be used to reduce postoperative opioid overprescribing in other regions or to address other public health efforts.

## Notes

Supplement 1. **eFigure 1.** STROBE Diagram Outlining Patients Included in Primary and Secondary Cohorts **eFigure 2.** Unadjusted Opioid Refill Rate, Unadjusted Opioid Satisfaction, Unadjusted Opioid Regret, and Unadjusted Opioid Quality of Life **eTable 1.** Comparison of Overall Sample With the Subset of the Sample That Responded to PRO Surveys **eTable 2.** Multivariable Regression Models for Satisfaction and Regret **eTable 3.** Multivariable Regression Models for Quality of Life and Pain

Supplement 2.
Data Sharing Statement

## References

[1] Share DA, Campbell DA, Birkmeyer N, . How a regional collaborative of hospitals and physicians in Michigan cut costs and improved the quality of care. Health Aff (Millwood). 2011;30(4):636-645. doi:10.1377/hlthaff.2010.0526 21471484

[2] Birkmeyer NJO, Share D, Campbell DA Jr, Prager RL, Moscucci M, Birkmeyer JD. Partnering with payers to improve surgical quality: the Michigan plan. Surgery. 2005;138(5):815-820. doi:10.1016/j.surg.2005.06.037 16291379

[3] Wandling MW, Minami CA, Johnson JK, . Development of a conceptual model for surgical quality improvement collaboratives: facilitating the implementation and evaluation of collaborative quality improvement. JAMA Surg. 2016;151(12):1181-1183. doi:10.1001/jamasurg.2016.2817 27604075 PMC8944346

[4] Guillamondegui OD, Gunter OL, Hines L, . Using the National Surgical Quality Improvement Program and the Tennessee Surgical Quality Collaborative to improve surgical outcomes. J Am Coll Surg. 2012;214(4):709-714. doi:10.1016/j.jamcollsurg.2011.12.012 22265639

[5] Campbell DA Jr, Kubus JJ, Henke PK, Hutton M, Englesbe MJ; The Michigan Surgical Quality Collaborative. The Michigan Surgical Quality Collaborative: a legacy of Shukri Khuri. Am J Surg. 2009;198(5)(suppl):S49-S55. doi:10.1016/j.amjsurg.2009.08.002 19874935

[6] Hendren S, Fritze D, Banerjee M, . Antibiotic choice is independently associated with risk of surgical site infection after colectomy: a population-based cohort study. Ann Surg. 2013;257(3):469-475. doi:10.1097/SLA.0b013e31826c4009 23059498

[7] Vu JV, Collins SD, Seese E, . Evidence that a regional surgical collaborative can transform care: surgical site infection prevention practices for colectomy in Michigan. J Am Coll Surg. 2018;226(1):91-99. doi:10.1016/j.jamcollsurg.2017.10.013 29111416

[8] Sutzko DC, Georgoff PE, Obi AT, Healy MA, Osborne NH. The association of venous thromboembolism chemoprophylaxis timing on venous thromboembolism after major vascular surgery. J Vasc Surg. 2018;67(1):262-271.e1. doi:10.1016/j.jvs.2017.06.087 28870681 PMC5741504

[9] Campbell DA, Englesbe MJ, Kubus JJ, . Accelerating the pace of surgical quality improvement: the power of hospital collaboration. Arch Surg. 2010;145(10):985-991. doi:10.1001/archsurg.2010.220 20956768

[10] Sheetz KH, Guy K, Allison JH, . Improving the care of elderly adults undergoing surgery in Michigan. J Am Geriatr Soc. 2014;62(2):352-357. doi:10.1111/jgs.12643 24428139

[11] Jaffe TA, Meka AP, semaan DZ, . Optimizing value of colon surgery in Michigan. Ann Surg. 2017;265(6):1178-1182. doi:10.1097/SLA.0000000000001880 27537537

[12] Bartels K, Mayes LM, Dingmann C, Bullard KJ, Hopfer CJ, Binswanger IA. Opioid use and storage patterns by patients after hospital discharge following surgery. PLoS One. 2016;11(1):e0147972. doi:10.1371/journal.pone.0147972 26824844 PMC4732746

[13] Bicket MC, Long JJ, Pronovost PJ, Alexander GC, Wu CL. Prescription opioid analgesics commonly unused after surgery: a systematic review. JAMA Surg. 2017;152(11):1066-1071. doi:10.1001/jamasurg.2017.0831 28768328 PMC5701659

[14] Bicket MC, White E, Pronovost PJ, Wu CL, Yaster M, Alexander GC. Opioid oversupply after joint and spine surgery: a prospective cohort study. Anesth Analg. 2019;128(2):358-364. doi:10.1213/ANE.0000000000003364 29677062

[15] Solouki S, Plummer M, Agalliu I, Abraham N. Opioid prescribing practices and medication use following urogynecological surgery. Neurourol Urodyn. 2019;38(1):363-368. doi:10.1002/nau.23867 30431173

[16] Lipari RN, Hughes A. How people obtain the prescription pain relievers they misuse. In: The CBHSQ Report. Substance Abuse and Mental Health Services Administration; 2017. Accessed April 16, 2020. https://www.ncbi.nlm.nih.gov/books/NBK424785/28252901

[17] Waljee JF, Li L, Brummett CM, Englesbe MJ. Iatrogenic opioid dependence in the United States: are surgeons the gatekeepers? Ann Surg. 2017;265(4):728-730. doi:10.1097/SLA.0000000000001904 27429023

[18] Brummett CM, Waljee JF, Goesling J, . New persistent opioid use after minor and major surgical procedures in US adults. JAMA Surg. 2017;152(6):e170504. doi:10.1001/jamasurg.2017.0504 28403427 PMC7050825

[19] Harbaugh CM, Lee JS, Hu HM, . Persistent opioid use among pediatric patients after surgery. Pediatrics. 2018;141(1):e20172439. doi:10.1542/peds.2017-2439 29203521 PMC7053700

[20] Vu JV, Howard RA, Gunaseelan V, Brummett CM, Waljee JF, Englesbe MJ. Statewide implementation of postoperative opioid prescribing guidelines. N Engl J Med. 2019;381(7):680-682. doi:10.1056/NEJMc1905045 31412184 PMC7160762

[21] Brown CS, Vu JV, Howard RA, . Assessment of a quality improvement intervention to decrease opioid prescribing in a regional health system. BMJ Qual Saf. 2021;30(3):251-259. doi:10.1136/bmjqs-2020-011295 32938775 PMC8056599

[22] Michigan OPEN. Prescribing recommendations. Accessed September 17, 2020. https://michigan-open.org/prescribing-recommendations/

[23] Mack KA, Zhang K, Paulozzi L, Jones C. Prescription practices involving opioid analgesics among Americans with Medicaid, 2010. J Health Care Poor Underserved. 2015;26(1):182-198. doi:10.1353/hpu.2015.0009 25702736 PMC4365785

[24] Centers for Disease Control and Prevention. Overdose deaths involving prescription opioids among Medicaid enrollees—Washington, 2004-2007. MMWR Morb Mortal Wkly Rep. 2009;58(42):1171-1175. 19875978

[25] Banks J, Hill C, Chi DL. Plan type and opioid prescriptions for children in Medicaid. Med Care. 2021;59(5):386-392. doi:10.1097/MLR.0000000000001504 33528236 PMC8026560

[26] Kaiser Family Foundation. Health insurance coverage of the total population. Published October 23, 2020. Accessed December 8, 2020. https://www.kff.org/other/state-indicator/total-population/

[27] Howard R, Fry B, Gunaseelan V, . Association of opioid prescribing with opioid consumption after surgery in Michigan. JAMA Surg. 2019;154(1):e184234. doi:10.1001/jamasurg.2018.4234 30422239 PMC6439853

[28] von Elm E, Altman DG, Egger M, Pocock SJ, Gøtzsche PC, Vandenbroucke JP; STROBE Initiative. The Strengthening the Reporting of Observational Studies in Epidemiology (STROBE) statement: guidelines for reporting observational studies. J Clin Epidemiol. 2008;61(4):344-349. doi:10.1016/j.jclinepi.2007.11.008 18313558

[29] Healy MA, Regenbogen SE, Kanters AE, . Surgeon variation in complications with minimally invasive and open colectomy: results from the Michigan Surgical Quality Collaborative. JAMA Surg. 2017;152(9):860-867. doi:10.1001/jamasurg.2017.1527 28614551 PMC5710462

[30] Swenson CW, Kamdar NS, Levy H, Campbell DA Jr, Morgan DM. Insurance type and major complications after hysterectomy. Female Pelvic Med Reconstr Surg. 2017;23(1):39-43. doi:10.1097/SPV.0000000000000325 27682744 PMC5161579

[31] Gammaitoni AR, Fine P, Alvarez N, McPherson ML, Bergmark S. Clinical application of opioid equianalgesic data. Clin J Pain. 2003;19(5):286-297. doi:10.1097/00002508-200309000-00002 12966254

[32] Brehaut JC, O’Connor AM, Wood TJ, . Validation of a decision regret scale. Med Decis Making. 2003;23(4):281-292. doi:10.1177/0272989X03256005 12926578

[33] Holmes-Rovner M, Kroll J, Schmitt N, . Patient satisfaction with health care decisions: the Satisfaction With Decision scale. Med Decis Making. 1996;16(1):58-64. doi:10.1177/0272989X9601600114 8717600

[34] Berkowitz R, Vu J, Brummett C, Waljee J, Englesbe M, Howard R. The impact of complications and pain on patient satisfaction. Ann Surg. 2021;273(6):1127-1134. doi:10.1097/SLA.0000000000003621 31663968 PMC7303925

[35] Schmocker RK, Cherney Stafford LM, Winslow ER. Satisfaction with surgeon care as measured by the Surgery-CAHPS survey is not related to NSQIP outcomes. Surgery. 2019;165(3):510-515. doi:10.1016/j.surg.2018.08.028 30322662 PMC6389404

[36] Elliott MN, Zaslavsky AM, Goldstein E, . Effects of survey mode, patient mix, and nonresponse on CAHPS hospital survey scores. Health Serv Res. 2009;44(2 Pt 1):501-518. doi:10.1111/j.1475-6773.2008.00914.x 19317857 PMC2677051

[37] Centers for Disease Control and Prevention. Prescription opioid data. Published March 13, 2020. Accessed April 26, 2021. https://www.cdc.gov/drugoverdose/deaths/prescription/index.html

[38] Lantz PM, House JS, Lepkowski JM, Williams DR, Mero RP, Chen J. Socioeconomic factors, health behaviors, and mortality: results from a nationally representative prospective study of US adults. JAMA. 1998;279(21):1703-1708. doi:10.1001/jama.279.21.1703 9624022

[39] Stone ML, LaPar DJ, Mulloy DP, . Primary payer status is significantly associated with postoperative mortality, morbidity, and hospital resource utilization in pediatric surgical patients within the United States. J Pediatr Surg. 2013;48(1):81-87. doi:10.1016/j.jpedsurg.2012.10.021 23331797 PMC3921619

[40] LaPar DJ, Bhamidipati CM, Mery CM, . Primary payer status affects mortality for major surgical operations. Ann Surg. 2010;252(3):544-550. doi:10.1097/SLA.0b013e3181e8fd75 20647910 PMC3071622

[41] Chapel JM, Ritchey MD, Zhang D, Wang G. Prevalence and medical costs of chronic diseases among adult Medicaid beneficiaries. *Am J Prev Med*. 2017;53(6S2):S143-S154. 10.1016/j.amepre.2017.07.019 PMC5798200 29153115

[42] Barnett ML, Sommers BD. A national survey of Medicaid beneficiaries’ experiences and satisfaction with health care. JAMA Intern Med. 2017;177(9):1378-1381. doi:10.1001/jamainternmed.2017.3174 28692734 PMC5818833

[43] Gilman BH, Kautter J. Impact of multitiered copayments on the use and cost of prescription drugs among Medicare beneficiaries. Health Serv Res. 2008;43(2):478-495. doi:10.1111/j.1475-6773.2007.00774.x 18370964 PMC2442369

[44] Reed M, Brand R, Newhouse JP, Selby JV, Hsu J. Coping with prescription drug cost sharing: knowledge, adherence, and financial burden. Health Serv Res. 2008;43(2):785-797. doi:10.1111/j.1475-6773.2007.00797.x 18370979 PMC2442368

[45] Simoni-Wastila L, Zuckerman IH, Shaffer T, Blanchette CM, Stuart B. Drug use patterns in severely mentally ill Medicare beneficiaries: impact of discontinuities in drug coverage. Health Serv Res. 2008;43(2):496-514. doi:10.1111/j.1475-6773.2007.00779.x 18370965 PMC2442367

[46] Wallace NT, McConnell KJ, Gallia CA, Smith JA. How effective are copayments in reducing expenditures for low-income adult Medicaid beneficiaries? experience from the Oregon Health Plan. Health Serv Res. 2008;43(2):515-530. doi:10.1111/j.1475-6773.2007.00824.x 18248405 PMC2442363

[47] Gatwood J, Gibson TB, Chernew ME, Farr AM, Vogtmann E, Fendrick AM. Price elasticity and medication use: cost sharing across multiple clinical conditions. J Manag Care Spec Pharm. 2014;20(11):1102-1107. doi:10.18553/jmcp.2014.20.11.1102 25351971 PMC10441015

[48] Kirsch M, Montgomery JR, Hu HM, . Association between insurance cost-sharing subsidy and postoperative opioid prescription refills among Medicare patients. Surgery. 2020;168(2):244-252. doi:10.1016/j.surg.2020.04.013 32505547 PMC8489972

