# Supplementary material for: Prescribed Opioid Dosages, Payer Type, and Self-Reported Outcomes After Surgical Procedures in Michigan, 2018-2020
Source: JAMA Netw Open. 2023 Jul 10;6(7):e2322581. doi: 10.1001/jamanetworkopen.2023.22581 (PMC10334228; doi:10.1001/jamanetworkopen.2023.22581)
Supplement: Supplement 1. — eFigure 1. STROBE Diagram Outlining Patients Included in Primary and Secondary Cohorts eFigure 2. Unadjusted Opioid Refill Rate, Unadjusted Opioid Satisfaction, Unadjusted Opioid Regret, and Unadjusted Opioid Quality of Life eTable 1. Comparison of Overall Sample With the Subset of the Sample That Responded to PRO Surveys eTable 2. Multivariable Regression Models for Satisfaction and Regret eTable 3. Multivariable Regression Models for Quality of Life and Pain [file jamanetwopen-e2322581-s001.pdf]

Supplementary Online Content

Breuler CJ, Shabet C, Delaney LD, et al. Prescribed opioid dosages, payer type, and self-reported outcomes after surgical procedures in Michigan, 2018-2020. *JAMA Netw Open*. 2023;6(7):e2322581. doi:10.1001/jamanetworkopen.2023.22581

eFigure 1. STROBE Diagram Outlining Patients Included in Primary and Secondary Cohorts
eFigure 2. Unadjusted Opioid Refill Rate, Unadjusted Opioid Satisfaction, Unadjusted Opioid Regret, and Unadjusted Opioid Quality of Life
eTable 1. Comparison of Overall Sample With the Subset of the Sample That Responded to PRO Surveys
eTable 2. Multivariable Regression Models for Satisfaction and Regret
eTable 3. Multivariable Regression Models for Quality of Life and Pain

This supplementary material has been provided by the authors to give readers additional information about their work.

© 2023 Breuler CJ et al. *JAMA Network Open*.

eFigure 1: **STROBE Diagram Outlining Patients Included in Primary and Secondary Cohorts.**

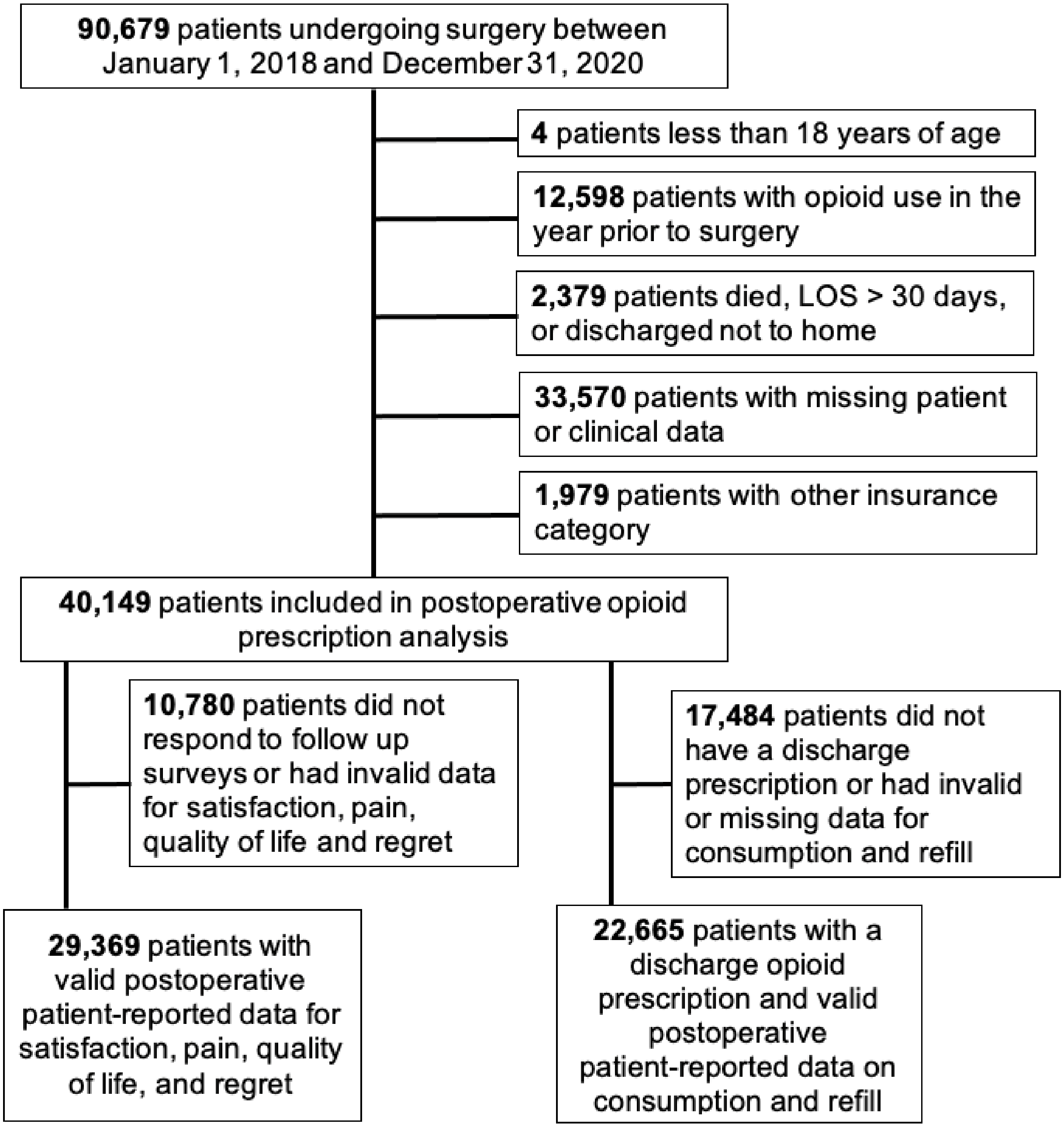

© 2023 Breuler CJ et al. *JAMA Network Open*.

eFigure2: (a) Unadjusted opioid refill rate (b) Unadjusted opioid satisfaction (c) unadjusted opioid regret (d) unadjusted opioid quality of life

(a)Unadjusted opioid refill rate

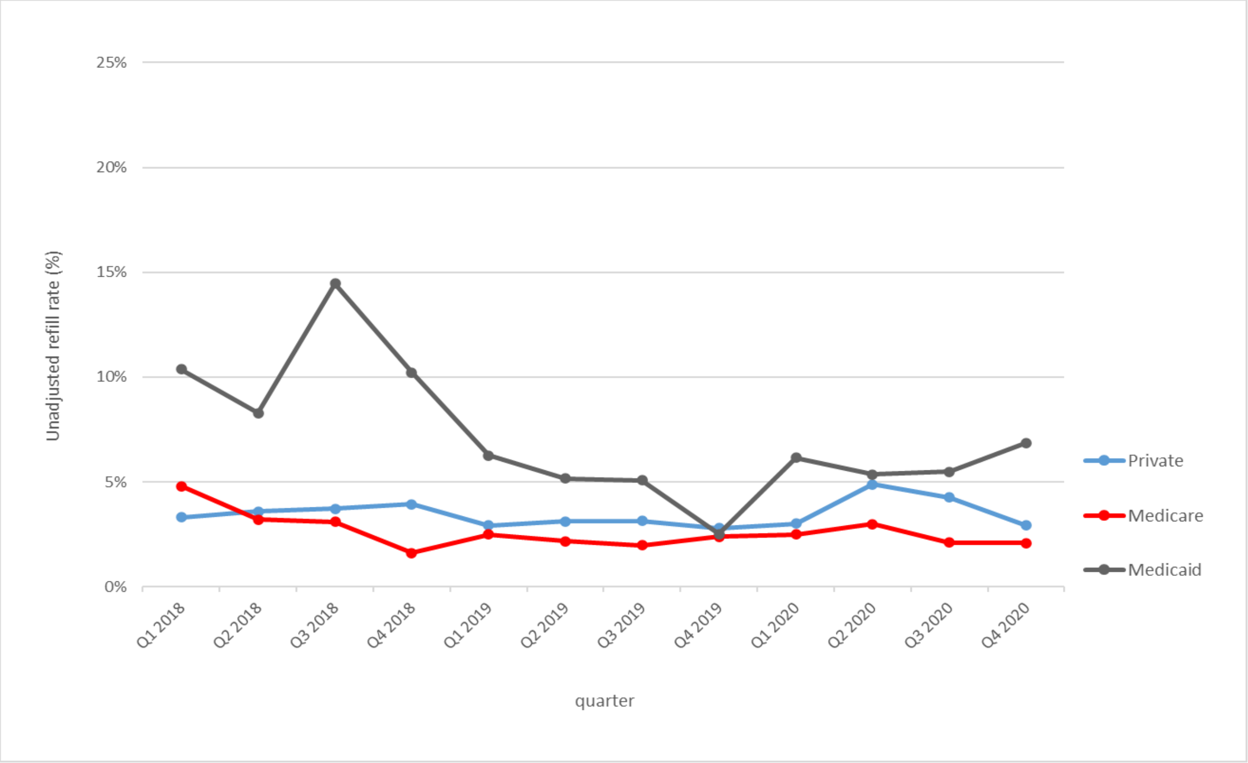

(b) Unadjusted opioid satisfaction

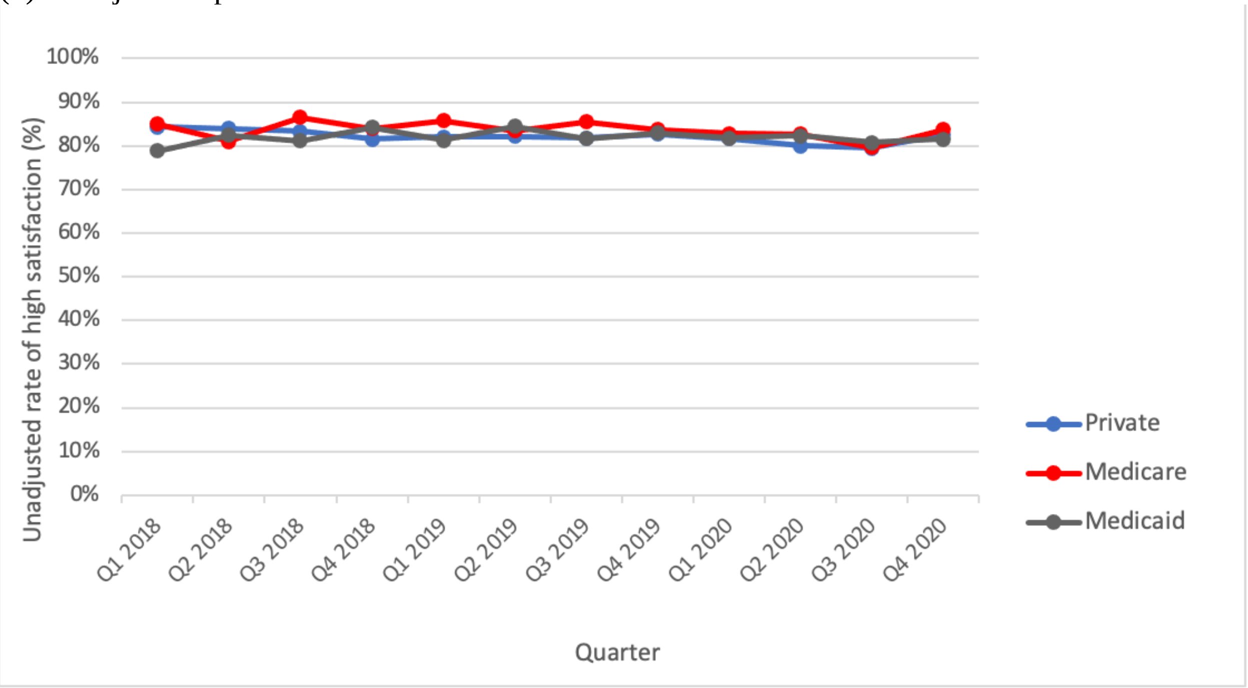

© 2023 Breuler CJ et al. *JAMA Network Open*.

(c) Unadjusted opioid regret

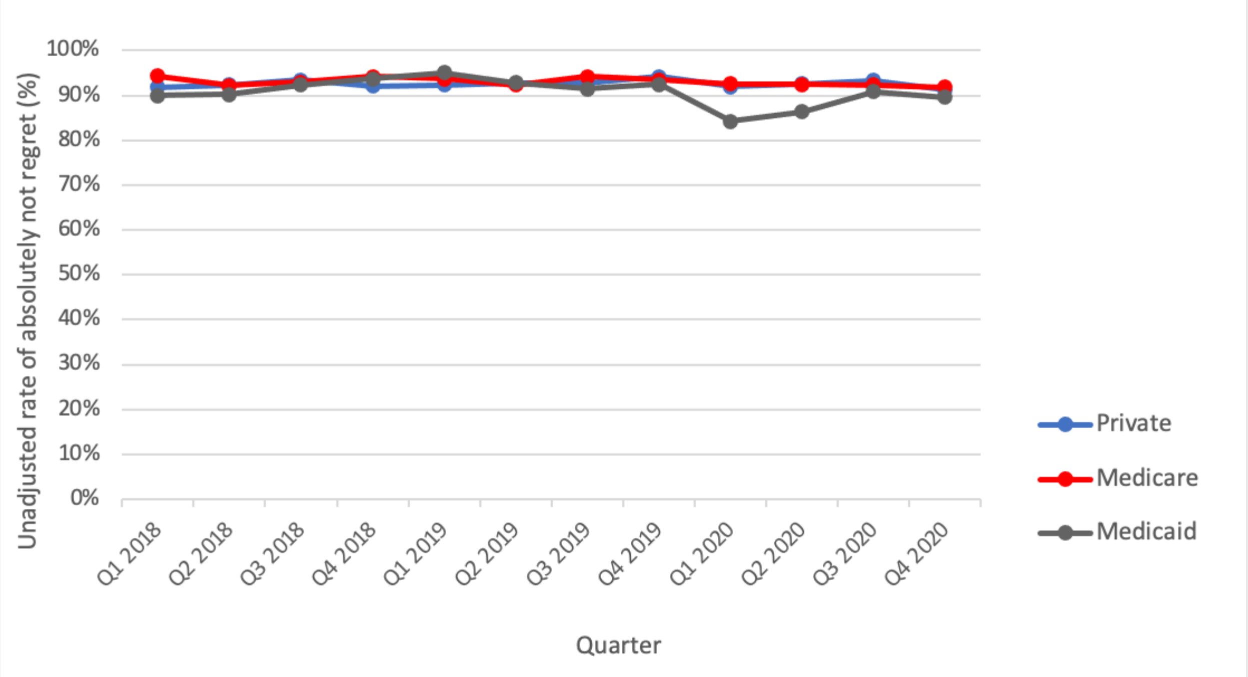

(d) Unadjusted opioid quality of life

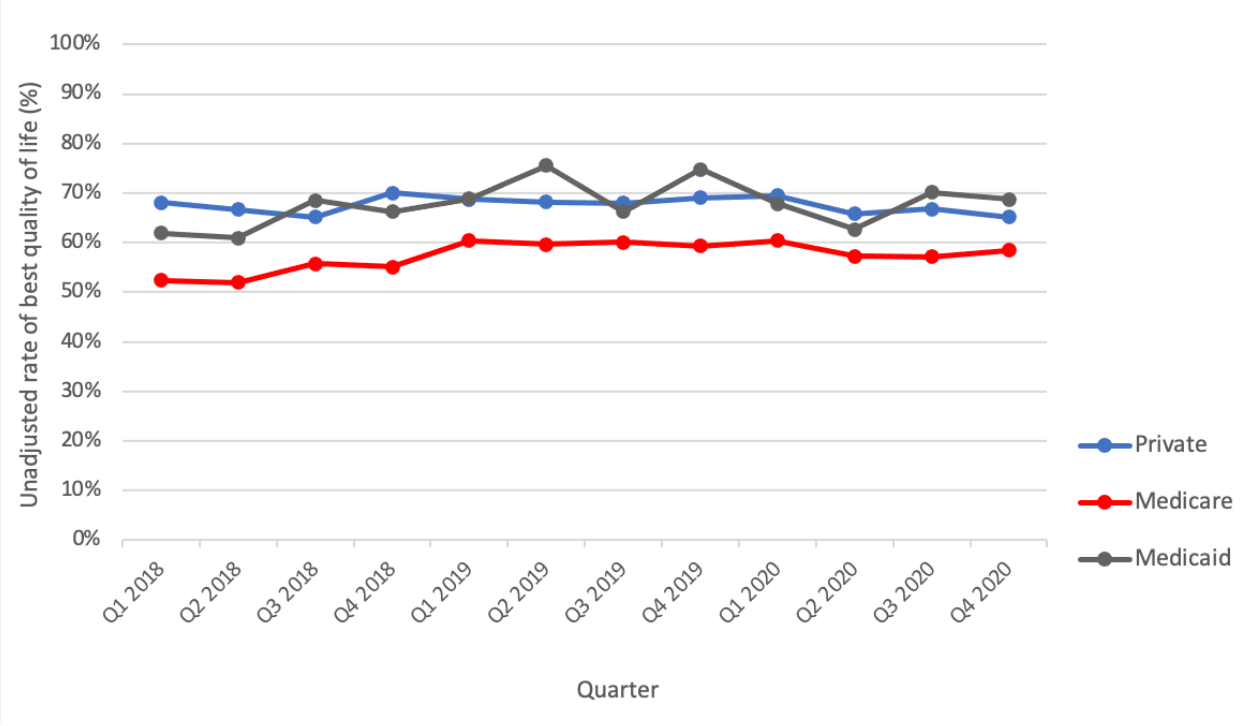

© 2023 Breuler CJ et al. *JAMA Network Open*.

eTable1 – Comparison of overall sample to the subset of the sample that responded to PRO surveys.

	**Overall sample (n=40,149)**		**PRO respondents (n=29,369)**		**P value**
	n	%	n	%	
Age					<0.001
18-29	3999	10.0	2587	8.8	
30-39	5614	14.0	3720	12.7	
40-49	7476	18.6	5170	17.6	
50-59	7984	19.9	5853	19.9	
60-64	4283	10.7	3293	11.2	
>=65	10793	26.9	8746	29.8	
Sex					0.129
Male	17228	42.9	12772	43.5	
Female	22921	57.1	16597	56.5	
Race					0.127
White, non-Hispanic	32926	82.0	23949	81.6	
Black, non-Hispanic	3146	7.8	2394	8.2	
Hispanic	956	2.4	659	2.2	
Other	3121	7.8	2367	8.1	
ASA Classification					<0.001
Class 1	3796	9.5	2719	9.3	
Class 2	23882	59.5	17074	58.1	
Class 3	11877	29.6	9118	31.1	
Class 4-5	594	1.5	458	1.6	
Obese	18535	46.2	13322	45.4	0.035
Cancer	2515	6.3	1984	6.8	0.009
Smoker	7214	18.0	4859	16.5	<0.001
Diabetes	4256	10.6	3245	11.1	0.060
Non-independent	137	0.3	91	0.3	0.475
Chronic Obstructive Pulmonary Disease	1295	3.2	974	3.3	0.505
Congestive Heart Failure	68	0.2	52	0.2	0.809
Hypertension	14488	36.1	11119	37.9	<0.001
Chronic Steroid Use	720	1.8	544	1.9	0.565
Dialysis	80	0.2	65	0.2	0.529
Admission Status: inpatient	18980	47.3	13821	47.1	0.577
Surgical Priority: Urgent/Emergent	9675	24.1	7041	24.0	0.707
Surgical Approach					0.841
Minimally Invasive	30315	75.5	22156	75.4	
Open	9834	24.5	7213	24.6	
Procedure Type					<0.001
Major Hernia	1739	4.3	1270	4.3	
Minor Hernia	11920	29.7	8945	30.5	
Laparoscopic Appendectomy	4490	11.2	3228	11.0	
Laparoscopic Cholecystectomy	11214	27.9	8255	28.1	
Laparoscopic Colectomy	1737	4.3	1400	4.8	
Open Colectomy	1127	2.8	846	2.9	
Vaginal Hysterectomy	2106	5.3	1494	5.1	
Laparoscopic Hysterectomy	4399	11.0	2887	9.8	
Total Abdominal Hysterectomy	1417	3.5	1044	3.6	

© 2023 Breuler CJ et al. *JAMA Network Open*.

eTable 2 – Multivariable regression models for satisfaction and regret (N=29,369)

Characteristic	High Satisfaction			No Regret		
	OR	95% CI	P	OR	95% CI	P
Insurance group (ref: Private)						
Medicare	1.09	0.89, 1.34	0.409	1.03	0.76, 1.39	0.848
Medicaid	0.92	0.74. 1.15	0.479	1.19	0.86, 1.64	0.290
Quarter	0.98	0.97, 1.00	0.007	0.99	0.98, 1.01	0.531
Insurance group*quarter interaction (ref: Private)						
Medicare	1.00	0.97, 1.02	0.723	0.98	0.95, 1.01	0.218
Medicaid	1.01	0.99, 1.04	0.346	0.96	0.92, 1.00	0.029
Age (ref: age 18-29)						
30-39	0.89	0.77, 1.01	0.077	0.91	0.75, 1.11	0.345
40-49	1.02	0.89, 1.16	0.790	0.93	0.77, 1.13	0.491
50-59	0.97	0.85, 1.10	0.629	0.93	0.77, 1.13	0.493
60-64	1.02	0.88, 1.18	0.828	1.11	0.89, 1.39	0.340
>=65	1.16	0.97, 1.38	0.100	1.34	1.04, 1.74	0.024
Sex						
Female	0.84	0.78, 0.91	<0.001	0.85	0.76, 0.95	0.004
Race (ref: White, non-Hispanic)						
Black, non-Hispanic	1.27	1.13, 1.43	<0.001	0.93	0.79, 1.09	0.357
Hispanic	1.46	1.16, 1.84	0.001	1.24	0.89, 1.73	0.193
Other	1.17	1.04, 1.32	0.007	0.76	0.66, 0.88	<0.001
ASA Classification (ref: Class 1)						
Class 2	0.90	0.80, 1.01	0.070	0.99	0.84, 1.17	0.920
Class 3	0.81	0.71, 0.92	0.002	0.90	0.75, 1.09	0.288
Class 4-5	0.86	0.65, 1.14	0.302	0.93	0.62, 1.39	0.721
Obese	1.05	0.98, 1.12	0.178	1.14	1.04, 1.26	0.006
Cancer	0.97	0.85, 1.11	0.637	1.30	1.06, 1.60	0.013
Smoker	0.97	0.90, 1.06	0.548	0.84	0.75, 0.95	0.004
Diabetes	0.96	0.87, 1.06	0.442	0.87	0.75, 1.00	0.053
Non-independent	0.95	0.56, 1.61	0.846	0.56	0.30, 1.05	0.070
Chronic Obstructive Pulmonary Disease	0.89	0.75, 1.05	0.180	0.97	0.76, 1.24	0.814
Congestive Heart Failure	1.03	0.50, 2.13	0.933	1.03	0.37, 2.89	0.959
Hypertension	0.97	0.90, 1.05	0.443	0.99	0.89, 1.10	0.836
Chronic Steroid Use	1.06	0.84, 1.32	0.637	0.73	0.55, 0.96	0.026
Dialysis	0.69	0.38, 1.25	0.219	0.63	0.29, 1.36	0.240
Admission Status: inpatient	0.88	0.80, 0.96	0.005	0.82	0.72, 0.94	0.004
Surgical Priority: Urgent/Emergent	1.06	0.95, 1.19	0.291	1.70	1.43, 2.01	<0.001
Surgical Approach: Open	0.93	0.83, 1.04	0.223	1.12	0.96, 1.31	0.147
Procedure Type (ref: Major Hernia)						
Minor Hernia	0.90	0.76, 1.07	0.239	0.99	0.78, 1.26	0.946
Laparoscopic Appendectomy	0.86	0.69, 1.07	0.169	1.59	1.14, 2.22	0.006
Laparoscopic Cholecystectomy	0.99	0.81, 1.20	0.912	1.10	0.84, 1.44	0.498
Laparoscopic Colectomy	0.96	0.75, 1.21	0.713	1.33	0.95, 1.88	0.099
Open Colectomy	0.79	0.63, 0.99	0.038	0.81	0.59, 1.13	0.215
Vaginal Hysterectomy	1.14	0.90, 1.44	0.275	1.38	1.00, 1.91	0.051
Laparoscopic Hysterectomy	1.11	0.90, 1.38	0.323	1.59	1.18, 2.14	0.003
Total Abdominal Hysterectomy	0.90	0.72, 1.12	0.342	1.11	0.81, 1.51	0.517

Legend: ASA indicates American Society of Anesthesiologists; COPD, chronic obstructive pulmonary disease; CHF, congestive heart failure.

© 2023 Breuler CJ et al. *JAMA Network Open*.

eTable 3 – Multivariable regression models for quality of life and pain (N=29,369)

Characteristic	Best QOL			Pain		
	OR	95% CI	P	OR	95% CI	P
Insurance group (ref: Private)						
Medicare	0.71	0.61, 0.83	<0.001	0.88	0.76, 1.02	0.087
Medicaid	0.83	0.69, 1.00	0.053	1.10	0.94, 1.30	0.238
Quarter	0.99	0.98, 1.00	0.086	1.02	1.01, 1.03	<0.001
Insurance group*quarter interaction (ref: Private)						
Medicare	1.03	1.01, 1.04	0.004	1.01	0.99, 1.02	0.386
Medicaid	1.02	1.00, 1.04	0.080	1.02	1.00, 1.04	0.038
Age (ref: age 18-29)						
30-39	0.86	0.76, 0.96	0.009	0.97	0.88, 1.07	0.588
40-49	0.86	0.76, 0.96	0.007	0.72	0.66, 0.80	<0.001
50-59	0.70	0.62, 0.78	<0.001	0.61	0.56, 0.67	<0.001
60-64	0.74	0.65, 0.84	<0.001	0.49	0.44, 0.54	<0.001
>=65	0.75	0.65, 0.87	<0.001	0.36	0.32, 0.41	<0.001
Sex						
Female	1.01	0.95, 1.07	0.795	1.33	1.26, 1.41	<0.001
Race (ref: White, non-Hispanic)						
Black, non-Hispanic	1.60	1.45, 1.77	<0.001	1.69	1.55, 1.83	<0.001
Hispanic	1.45	1.21, 1.73	<0.001	1.21	1.04, 1.40	0.012
Other	1.30	1.19, 1.43	<0.001	1.07	0.99, 1.16	0.082
ASA Classification (ref: Class 1)						
Class 2	0.86	0.78, 0.95	0.002	1.09	1.01, 1.18	0.028
Class 3	0.68	0.61, 0.76	<0.001	1.25	1.14, 1.37	<0.001
Class 4-5	0.53	0.43, 0.66	<0.001	1.17	0.95, 1.43	0.139
Obese	0.98	0.93, 1.03	0.408	0.97	0.93, 1.02	0.256
Cancer	0.74	0.67, 0.82	<0.001	0.88	0.80, 0.97	0.012
Smoker	0.93	0.87, 1.00	0.047	1.26	1.19, 1.34	<0.001
Diabetes	0.93	0.85, 1.00	0.060	0.99	0.92, 1.07	0.870
Non-independent	0.85	0.56, 1.30	0.452	0.78	0.52, 1.15	0.208
Chronic Obstructive Pulmonary Disease	0.84	0.74, 0.96	0.012	1.10	0.97, 1.25	0.139
Congestive Heart Failure	1.10	0.63, 1.92	0.746	1.66	0.97, 2.84	0.065
Hypertension	0.95	0.90, 1.01	0.102	0.96	0.91, 1.02	0.162
Chronic Steroid Use	0.89	0.74, 1.06	0.185	1.08	0.92, 1.27	0.342
(Dialysis)	0.66	0.40, 1.10	0.109	1.16	0.72, 1.88	0.534
Admission Status: inpatient	0.96	0.89, 1.03	0.277	1.13	1.06, 1.21	<0.001
Surgical Priority: Urgent/Emergent	1.10	1.01, 1.20	0.037	0.83	0.77, 0.90	<0.001
Surgical Approach: Open	0.88	0.81, 0.96	0.004	1.03	0.95, 1.11	0.469
Procedure Type (ref: Major Hernia)						
Minor Hernia	0.92	0.81, 1.06	0.243	1.26	1.12, 1.43	<0.001
Laparoscopic Appendectomy	1.00	0.83, 1.19	0.968	0.86	0.74, 1.01	0.073
Laparoscopic Cholecystectomy	0.92	0.79, 1.07	0.286	0.78	0.68, 0.90	<0.001
Laparoscopic Colectomy	0.77	0.64, 0.93	0.006	1.04	0.88, 1.24	0.649
Open Colectomy	0.70	0.58, 0.84	<0.001	1.38	1.16, 1.64	<0.001
Vaginal Hysterectomy	0.95	0.79, 1.14	0.568	0.64	0.54, 0.75	<0.001
Laparoscopic Hysterectomy	0.96	0.81, 1.13	0.604	0.62	0.53, 0.73	<0.001
Total Abdominal Hysterectomy	0.97	0.80, 1.16	0.724	1.35	1.15, 1.59	<0.001

Legend: ASA indicates American Society of Anesthesiologists; COPD, chronic obstructive pulmonary disease; CHF, congestive heart failure

© 2023 Breuler CJ et al. *JAMA Network Open*.
