# Supplementary material for: Prescribed Opioid Dosages, Payer Type, and Self-Reported Outcomes After Surgical Procedures in Michigan, 2018-2020
Source: JAMA Netw Open. 2023 Jul 10;6(7):e2322581. doi: 10.1001/jamanetworkopen.2023.22581 (PMC10334228; doi:10.1001/jamanetworkopen.2023.22581)
Supplement: Supplement 2. — Data Sharing Statement [file jamanetwopen-e2322581-s002.pdf]

Data Sharing Statement

Breuler. Prescribed Opioid Dosages, Payer Type, and Self-Reported Outcomes After Surgical Procedures in Michigan, 2018-2020. *JAMA Netw Open*. Published July 10, 2023. doi:10.1001/jamanetworkopen.2023.22581

Data
Data available: No
